# The role of perfusion CT in identifying stroke mimics in the emergency room: a case of status epilepticus presenting with perfusion CT alterations

**DOI:** 10.1186/1865-1380-5-4

**Published:** 2012-01-20

**Authors:** Waldo R Guerrero, Haitham Dababneh, Stephan Eisenschenk

**Affiliations:** 1 Department of Neurology, College of Medicine, University of Florida, 1601 Archer Road Gainesville, 32610-0236 FL, USA

## Abstract

Emergency medicine physicians are often faced with the challenging task of differentiating true acute ischemic strokes from stroke mimics. We present a case that was initially diagnosed as acute stroke. However, perfusion CT and EEG eventually led to the final diagnosis of status epilepticus. This case further asserts the role of CT perfusion in the evaluation of patients with stroke mimics in the emergency room setting.

## Background

The differentiation between stroke and seizure can be a clinically arduous task for both emergency medicine physicians and neurologists [1,2]. Patients with diseases that mimic stroke account for one-fifth of patients with brain attacks [1]. Imaging may therefore be critical in making a diagnosis in the acute setting. Seizure is one condition that can mimic a stroke. Commonly, patients with Todd's paralysis or those with nonconvulsive status epilepticus can be clinically indistinct from those with acute stroke. Further complicating the clinical scenario, seizure may also be a presenting sign of stroke [3]. Recently the time frame for standard treatment of acute stroke with IV tissue plasminogen activator was expanded from 3 h to 4.5 h from ictus onset [4]. Although this extension of time is supported by the American Heart Association, it is not FDA approved and comes with a different set of relative contraindications. Intravenous thrombolytics are not without the risk of complications, including intracranial hemorrhage [5]. Non-contrast CT (NCCT) of the head is the current gold standard in excluding intracranial hemorrhage prior to administration of intravenous thrombolysis. However, NCCT has a limited role in differentiating those patients with stroke from those with seizure. Although current guidelines advocate only NCCT as the imaging modality of choice in the initial evaluation of acute stroke, this case illustrates the importance of CT perfusion studies in the radiographic evaluation of brain attack patients in order to avoid misdiagnosis and inadvertent treatment of non-stroke patients with thrombolytic therapy. Furthermore, whereas hypoperfusion related to strokes has been widely investigated by CT-perfusion imaging [6,7], this case demonstrates the hyperperfusion state often seen on perfusion CT in emergency room patients with epilepsy. We describe an interesting case of a patient presenting to the Shands Hospital at the University of Florida emergency room with a homonymous hemianopsia and alterations on perfusion CT related to hyperglycemia-induced occipital status epilepticus.

## Case presentation

**Figure 1:**
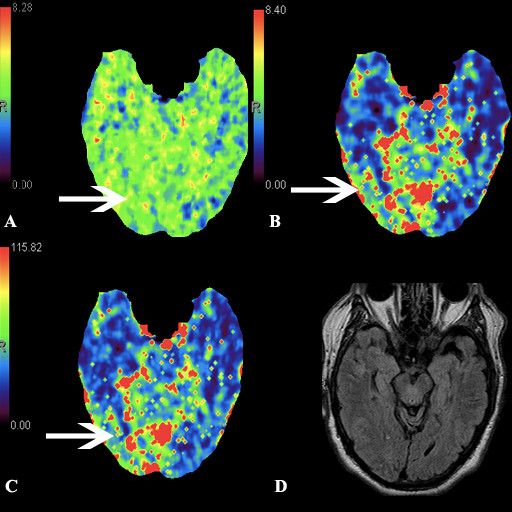
**CT perfusion study demonstrating (**a**) shortened mean transit time in the right occipital territory (*arrow*)**. Corresponding area of increased cerebral blood volume (**b**) and increased cerebral blood flow (**c**). FLAIR MRI brain (**d**) without evidence of infarct.

A 72-year-old man with a past medical history significant for diabetes mellitus type 2 presented to the Shands Hospital at the University of Florida emergency room with sudden onset of visual changes. The patient had noted that he would miss objects when reaching for them at home. He also noted black and red spots and prisms in his vision. There was no previous history of seizures. His risk factors for stroke included his age, diabetes type 2, as well as tobacco use. The NIH Stroke Scale score on presentation was 2 with a complete left homonymous hemianopsia. He presented within 3 h of symptom onset, and perfusion CT was obtained per the stroke protocol at our institution. Non-contrast CT did not reveal any early signs of stroke or hemorrhage. The CT angiogram was unremarkable. Perfusion CT demonstrated an area of shortened mean transit time (MTT), increased cerebral blood volume (CBV), and increased cerebral blood flow (CBF) in the right occipital territory (Figure 1). Given the patient's left homonymous hemianopsia, the findings on perfusion CT were interpreted as a hyperperfusion phenomenon. The differential diagnosis included epileptiform activity as well as other pathologies not limited to glioma and misery perfusion syndrome. Thus, an EEG was obtained, and it confirmed nonconvulsive seizure activity from the right parieto-occipital quadrant. This patient was eligible for intravenous thrombolysis given the neurological findings and NIHSS of 2; however, the constellation of the above findings led to our clinical decision not to administer thrombolytics in the setting of a seizure diagnosis. The patient was loaded with Ativan, Fosphenytoin, and Depakote in order to stop the seizures. Once the seizures were controlled, the patient's neurological exam normalized. Follow-up FLAIR MRI brain imaging did not reveal evidence of stroke (Figure 1).

## Discussion

Stroke mimics account for 5-30% of "brain attacks." Of those patients receiving thrombolytic therapy in the European Cooperative Acute Stroke Study II (ECASS II), 17% were ultimately shown to not have had strokes [8]. Common conditions such as migraine, epilepsy with and without Todd's paralysis, hypoglycemia, and sinus thrombosis can often mimic stroke [9,10]. Unfortunately, NCCT is not a sensitive radiographic tool in detecting stroke because parenchymal changes do not usually appear early in the course of acute stroke [1,11]. MRI would offer good accuracy and sensitivity in such cases [12]. However, it is often not utilized because of its decreased availability in contrast to the short acquisition time and wide availability for NCCT in the emergency room setting.

There are data supporting the use of CT perfusion in acute stroke management [11]. Relative MTT and absolute CBV are CT perfusion parameters that help define areas of infarct from areas of penumbra [6]. Its use has also been investigated for the diagnosis of seizures [13,14]. Hauf et al. demonstrated that perfusion CT is a useful tool in accelerating the diagnosis of nonconvulsive status epilepticus with a sensitivity of 78% [15]. In this case, cortical hyperperfusion was observed as reflected by a decrease in mean transient time (MTT) and a concomitant increase in cerebral blood volume (CBV) and flow (CBF) (Figure 1). This is compatible with previous data demonstrating increased CBV and CBF values in the seizure onset zone as well as in the regions with ictal spread [13]. This hyperperfusion state during the ictal state has also been shown with SPECT and f-MRI in patients with focal epilepsy [16,17].

CT perfusion has the advantages of routine availability, short acquisition time, and quantitative results. This case further supports the role of CT perfusion in the emergency room setting when assessing stroke patients for thrombolytics. Although patients with stroke mimics infrequently receive thrombolytics and their treatment generally does not lead to harmful complications [18], CT perfusion may spare patients with status epilepticus from the misguided treatment of intravenous thrombolysis. PCT may also qualify as a complementary diagnostic tool in patients presenting to the emergency room with altered mental status in which stroke is also a consideration for etiology.

## Conclusions

In summary, perfusion CT can serve an important role in differentiating acute stroke from an unusual presentation of status epilepticus in the emergency room setting.

## Abbreviations

NCCT: non-contrast CT head, MTT: mean transit time. CBV: cerebral blood volume, CBF: cerebral blood flow, SPECT: single-photon emission computed tomography, f-MRI: functional magnetic resonance imaging.

## Consent

Written informed consent was obtained from the patient for publication of this case report and any accompanying images. A copy of the written consent is available for review by the Editor-in-Chief of this journal.

## Competing interests

The authors declare that they have no competing interests.

## Authors' contributions

WG drafted the manuscript and collected the images and figure utilized in this manuscript. HD edited the manuscript and assisted in CT perfusion image interpretation. SE edited the manuscript as well as supervised. All authors read and approved the final manuscript.

## Authors' information

WG and HD are both fourth year Neurology residents at the University of Florida. SE is Associate Professor of Neurology, Clinical Director, Adult Neurology Comprehensive Epilepsy Program, and Medical Director, UF & Shands Epilepsy Monitoring Unit.

## Acknowledgements

Publication of this article was funded in part by the University of Florida Open-Access Publishing Fund.
